# Acupuncture for functional dyspepsia: study protocol for a two-center, randomized controlled trial

**DOI:** 10.1186/1745-6215-15-89

**Published:** 2014-03-22

**Authors:** Gajin Han, Seok-Jae Ko, Jae-Woo Park, Jinsung Kim, Inkwon Yeo, Hyejung Lee, Song-Yi Kim, Hyangsook Lee

**Affiliations:** 1 Department of Gastroenterology, College of Korean Medicine, Kyung Hee University, Kyung Hee dae-ro 26, Dongdaemun-gu, Seoul 130-701, Republic of Korea; 2 Department of Statistics, Sookmyung Women’s University, Cheongpa-ro 47-gil 100, Youngsan-gu, Seoul 140-742, Republic of Korea; 3 Acupuncture and Meridian Science Research Center, College of Korean Medicine, Kyung Hee University, Kyung Hee dae-ro 26, Dongdaemun-gu, Seoul 130-701, South Korea

**Keywords:** Acupuncture, Functional dyspepsia, Ghrelin, Randomized controlled trial, Waitlist

## Abstract

**Background:**

Functional dyspepsia (FD) is a common health problem currently without any optimal treatments. Acupuncture has been traditionally sought as a treatment for FD. The aim of this study is to investigate whether acupuncture treatment helps improve symptoms of FD.

**Methods/design:**

A two-center, randomized, waitlist-controlled trial will be carried out to evaluate whether acupuncture treatment improves FD symptoms. Seventy six participants aged 18 to 75 years with FD as diagnosed by Rome III criteria will be recruited from August 2013 to January 2014 at two Korean Medicine hospitals. They will be randomly allocated either into eight sessions of partially individualized acupuncture treatment over 4 weeks or a waitlist group. The acupuncture group will then be followed-up for 3 weeks with six telephone visits and a final visit will be paid at 8 weeks. The waitlist group will receive the identical acupuncture treatment after a 4-week waiting period. The primary outcome is the proportion of responders with adequate symptom relief and the secondary outcomes include Nepean dyspepsia index, EQ-5D, FD-related quality of life, Beck’s depression inventory, state-trait anxiety inventory questionnaire, and level of ghrelin hormone. The protocol was approved by the participating centers’ Institutional Review Boards.

**Discussion:**

Results of this trial will help clarify not only whether the acupuncture treatment is beneficial for symptom improvement in FD patients but also to elucidate the related mechanisms of how acupuncture might work.

**Trial registration:**

ClinicalTrials.gov Identifier: NCT01921504.

## Background

Functional dyspepsia (FD) is a functional gastrointestinal disorder without any structural lesion [1]. It is reported that the prevalence of FD ranges from 8% to 23% in Asia [2], and in particular, FD is claimed to affect 25% of the South Korean population [3]. Though FD is not a life-threatening disease, FD patients suffer from a poor quality of life (QoL), which is regarded as an economic burden on society [4].

Although various treatments for FD, such as proton pump inhibitors, prokinetic agents, tricyclic antidepressants, and antinociceptive agents are available [5-9], many FD patients turn to other complementary and alternative therapies largely due to a lack of satisfactory relief by these treatments [10].

Acupuncture treatment is one of the most sought-after therapeutic modalities in complementary and alternative medicine. It has been frequently used to treat symptoms of FD [11] largely based on the following rationales: firstly, acupuncture is well known to relieve pain in various conditions [12] and may help reduce epigastric pain or burning sensation [4]; secondly, previous studies have shown that manual acupuncture and electro-acupuncture modulates gastric/duodenal motility through the activation of sympathetic efferent nerve fibers or vagal nerve fibers [13]; finally, since patients with FD have been reported to have higher levels of psychological distress or depression than healthy subjects [14,15], the anxiolytic or antidepressant effects of acupuncture have been utilized to minimize worsening of FD symptoms [16-18].

However, previous studies have reported inconsistent findings of the clinical benefit of acupuncture treatment for FD [4,19]. Nevertheless, these studies have been criticized for adopting relatively sub-optimal acupuncture treatment, i.e., three times a week for 2 weeks [19,20], or testing a less generalizable treatment regimen, i.e., daily acupuncture treatment for 4 weeks [4]. According to a systematic review by Zhu [21], the acupuncture treatment for FD lacks high-quality evidence. Hence, it was addressed that randomized clinical trials corresponding with the CONSORT statement and STandards for Reporting Interventions in Clinical Trials of Acupuncture (STRICTA) recommendations have to be implemented to evaluate the efficacy of acupuncture for FD. In addition, considering the questions which have been raised so far on the adequacy of various sham acupuncture controls [22,23], care should be taken before simply adopting any available sham devices or procedures in the early phase.

Given that FD is now regarded as a complex disorder of which the pathophysiological mechanisms are inconclusive [24], various factors are likely to come into play. Among them, ghrelin, an orexigenic peptide, has been shown to play an important role in gastric motility, food intake, and potential gastroprotection against acute gastric mucosal injury [25,26]. However, it is yet to be studied whether ghrelin is associated with the mechanisms of acupuncture treatment in patients with FD [27,28].

Taken together, we felt the need to properly test whether acupuncture treatment would help symptom improvement in FD patients and, subsequently, the related mechanism. Therefore, we propose a two-center, randomized, waitlist-controlled trial investigating the effectiveness of acupuncture for symptom improvement in patients with FD and to assess whether the change in ghrelin secretion is associated with acupuncture treatment.

## Methods/design

### Objectives

The aims of this study are to: i) assess the effect of acupuncture treatment on patients with FD in comparison with waitlist group with respect to adequate symptom relief and ii) to elucidate whether ghrelin level is associated with clinical effect of acupuncture.

### Hypothesis

We hypothesize that eight sessions of acupuncture treatment over 4 weeks will improve symptoms of FD.

### Design

**Figure 1:**
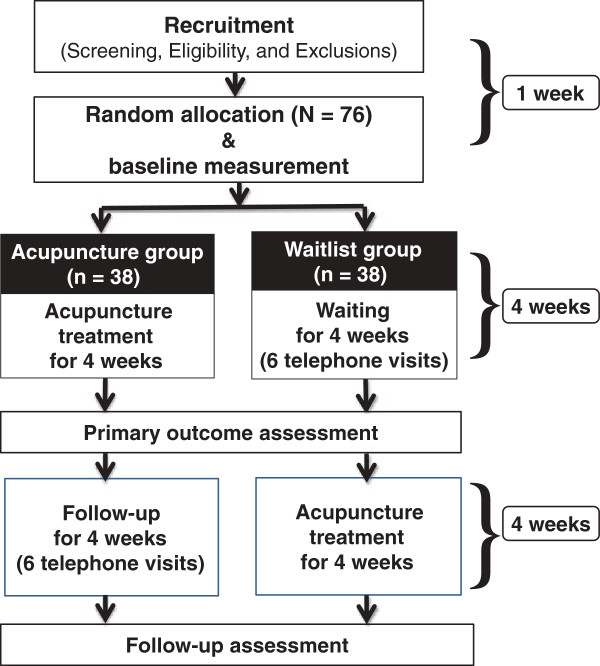
Flow of the study.

A two-center, randomized, waitlist-controlled trial will be conducted at the Kyung Hee University Korean Medicine Hospital and Kyung Hee University Hospital at Gangdong in Seoul, Korea from August 2013 to January 2014. The flow of the entire trial is shown in Figure 1.

### Inclusion and exclusion criteria

#### Inclusion criteria

Participants who meet the following criteria will be included:

i) Individuals between the ages of 18 and 75 years, able to read and write Korean language

ii) Individuals who meet the Rome III FD criteria

iii) Individuals who check more than four points on the visual analogue scale (VAS; 0, no symptom disturbance at all; 10, very severe) for dyspeptic symptoms

iv) Individuals who have normal esophagogastroduodenoscopy results within a year and have been diagnosed with FD by a specialist consultation

v) Individuals who receive no other treatments during the study

vi) Individuals who voluntarily agree with a study protocol and sign a written informed consent

#### Exclusion criteria

Participants who report any of the following will be excluded:

i) Individuals who have peptic ulcer or gastroesophageal reflux disease confirmed by esophagogastroduodenoscopy

ii) Individuals who have obvious signs of irritable bowel syndrome

iii) Individuals who have alarm symptoms (weight loss, black or tar stool, or dysphagia)

iv) Individuals who have serious structural disease (disease of heart, lung, liver or kidney) or mental illness

v) Individuals who have had surgery related with the gastrointestinal tract, except for appendectomy more than six months ago

vi) Individuals who are pregnant or breastfeeding

vii)  Individuals who are taking drugs which might affect the gastrointestinal tract; a minimum wash-out period of two weeks is required before participating in the trial

viii)  Individuals who are HIV-positive

ix) Individuals who have a problem of malabsorption or maldigestion

x) Individuals who have difficulties in attending the trial (e.g., paralysis, serious mental illness, dementia, drug addiction, time constraint, severe disorder in vision or hearing, illiteracy)

xi) Individuals unwilling to sign the informed consent

xii)  Individuals with other diseases that could interfere with acupuncture treatment, e.g., clotting disorders or leukopenia, pace-maker, epilepsy, or anticoagulant therapy

### Recruitment

Patients who have participated in our previous studies will be contacted to ask if they are interested. Advertisements will be put on notice boards and homepages of the hospitals, advertising boards on the subway trains, and local newspapers. Obtaining consent will be carried out by the investigators with the principle of informed consent, which consists of information, decisional capacity, and voluntarism. The participants will sign two written informed consent forms, general consent form and additional consent provisions for collection and use of their human biologic material, and go through the screening test. The model consent form and other related documentation given to participants can be provided upon request.

### Randomization

After signing an informed consent and undergoing the screening test, the participants in each hospital will be randomly assigned to one of the two groups using block randomization. Random numbers will be generated using the PROC PLAN of SAS 9.2 (SAS Institute Inc., Cary, NC, USA) by an independent statistician and sent to the Acupuncture and Meridian Science Research Center (AMSRC) at Kyung Hee University where the each randomization number will be put in a sequentially numbered opaque envelope and remain sealed by an administrative staff who is otherwise not involved in the study.

Once a participant passes the screening test, the investigator will call the AMSRC staff who is responsible for this randomization process to give her the hospital name, and the initial and screening number of the participant. The AMSRC staff will then immediately fax a document containing a unique random number and participant group allocation. This feedback document will be kept in the trial master file.

### Intervention

#### Acupuncture treatment

Detailed information on acupuncture treatment is summarized based on the revised STRICTA recommendations [29] in Table 1. The acupuncture treatment procedures were designed to reflect the real clinical setting.

**Table 1:** **Acupuncture treatment details based on the STRICTA 2010 checklist**[29]

**Item**	**Detail**
**1. Acupuncture rationale**	**1a) Style of acupuncture**
	- Manual acupuncture based on traditional meridian theory.
	**1b) Reasoning for treatment provided, based on historical context, literature sources, and/or consensus methods, with references where appropriate**
	- Partially-individualized manual acupuncture treatments based on the traditional meridian theory, clinical experience, and consensus by the experts in acupuncture and FD.
	**1c) Extent to which treatment was varied**
	- Partially-individualized acupuncture treatment, i.e., fixed points plus optional points according to symptoms.
**2. Details of needling**	**2a) Number of needle insertions per subject per session (mean and range where relevant)**
	- From 9 to 19.
	**2b) Names (or location if no standard name) of points used (uni/bilateral)**
	- Nine fixed points: LI4, ST36, LR3, and SP4 (bilateral) and CV12 (unilateral).
	- Optional points according to individual symptoms: GB21, SI14, PC6, EX-HN5, and ST34 (bilateral).
	**2c) Depth of insertion, based on a specified unit of measurement, or on a particular tissue level**
	- From 5 to 30 mm.
	**2d) Response sought (e.g., de qi or muscle twitch response)**
	- ‘De qi’ sensation.
	**2e) Needle stimulation (e.g., manual, electrical)**
	- Manual stimulation: needle rotation with thumb and index fingers at 3 Hz.
	**2f) Needle retention time**
	- Fifteen minutes.
	**2g) Needle type (diameter, length, and manufacturer or material)**
	- A sterilized stainless steel needle (0.25?×?40 mm, Dongbang Acupuncture Inc., Bundang, Sungnam, Korea).
**3. Treatment regimen**	**3a) Number of treatment sessions**
	- Eight treatment sessions in both acupuncture and waitlist groups.
	**3b) Frequency and duration of treatment sessions**
	- Twice weekly for 4 weeks, 15 minutes for each session.
**4. Other components of treatment**	**4a) Details of other interventions administered to the acupuncture group (e.g., moxibustion, cupping, herbs, exercises, lifestyle advice)**
	- No other interventions during the study period allowed.
	**4b) Setting and context of treatment, including instructions to practitioners, and information and explanations to patients**
	- University hospitals.
	- Participants will be informed about acupuncture treatment in the study as follows: “In this study, acupoint for FD will be used based on traditional Korean medicine textbook and FD-related reports.”
**5. Practitioner background**	**5) Description of participating acupuncturists (qualification or professional affiliation, years in acupuncture practice, other relevant experience)**
	- Korean Medicine Doctors who have a license and at least 3 years of experience in gastrointestinal disorders. They went through 10 hours of training and simulation to ensure that they are able to provide identical acupuncture treatment in accordance with a pre-defined protocol.
**6. Control or comparator interventions**	**6a) Rationale for the control or comparator in the context of the research question, with sources that justify this choice**
	- A waitlist group will be adopted as a first step before we move on to a sham controlled trial.
	**6b) Precise description of the control or comparator. If sham acupuncture or any other type of acupuncture-like control is used, provide details as for items 1 to 3 above.**
	- Participants in the waitlist will not receive acupuncture treatment after randomization for 4 weeks, during which period, there will be six telephone visits. After this waiting period, they then will receive twice weekly acupuncture sessions for 4 weeks in the same manner as in the acupuncture group.

CV, Conception Vessel; EX-HN, Extra acupoint of Head and Neck; FD, functional dyspepsia; GB, Gallbladder; LI, Large Intestine; LR, Liver; PC, Pericardium; SI, Small Intestine; SP, Spleen; ST, Stomach; STRICTA, STandards for Reporting Interventions in Clinical Trials of Acupuncture.

In this trial, partially-individualized manual acupuncture treatments based on the traditional meridian theory and consensus by the experts in acupuncture and FD, will be given. The number of inserted needles per participant per session ranges from 9 to 19. The acupuncture points consist of basic points and optional points. All participants will be given needling at nine fixed points: Large Intestine 4, Stomach (ST) 36, Liver 3, and Spleen 4 bilaterally, and Conception Vessel 12 unilaterally. The optional points include Gallbladder (GB) 21, Small Intestine (SI) 14, Pericardium (PC) 6, Extra acupoint of Head and Neck (EX-HN) 5, and ST 34. These optional points will be selected according to the participant’s symptoms such as headache (EX-HN5), shoulder or back pain (GB21, SI14), nausea and/or vomiting (PC6), and heartburn or epigastric pain (ST34). Two additional Ah-Shi points in the medial scapular region will be allowed in case of shoulder or back pain.

The questions about symptoms related to optional points will be asked to participants by the clinical research coordinator to minimize the bias induced by contact between doctor and participant.

A sterilized stainless steel needle (40 mm length and 0.25 mm diameter; Dongbang Acupuncture Inc., Bundang, Sungnam, Korea) will be used. The depth of needle insertion will be from 5 mm to 30 mm and needle manipulation to achieve ‘de qi’ sensation will be performed. The acupuncture needles will be retained for 15 min.

All acupuncture treatments will be provided by the same Korean Medicine Doctor at each hospital, who will have undergone 10 hours of training and simulation to ensure that they are able to provide identical acupuncture treatment in accordance with a pre-defined protocol.

#### Acupuncture group

The participants in the acupuncture group will receive eight acupuncture sessions over 4 weeks. After the treatment period, they will be followed-up for 3 weeks with six telephone visits to collect adequate relief data and they will pay a final visit at 8 weeks. No other interventions during the study period will be allowed and a diary will be collected.

#### Waitlist group

The participants in the waitlist group will not receive acupuncture treatment after randomization for 4 weeks, during which period there will be six telephone visits once or twice a week. After this waiting period, they will then receive twice weekly acupuncture sessions for 4 weeks in the same manner as in the acupuncture group. No other interventions during the study period will be allowed and a diary will be collected.

### Outcome measures

A nurse in each center who is otherwise involved in the study will give the participants a set of outcome measures to complete in addition to the primary outcome measure; its schedule is summarized in Table 2.

**Table 2:** Study schedule for data collection

**Measures**	**Baseline**	**1-week**		**2-week**		**3-week**		**4-week**		**5-week**		**6-week**		**7-week**		8-week*
Sociodemographic data	✔															
Adequate relief^†^ for the acupuncture group		✔	✔	✔	✔	✔	✔	✔	✔	✔	✔	✔	✔	✔	✔	✔
Adequate relief^§^ for the waitlist group		✔	✔	✔	✔	✔	✔	✔	✔	✔	✔	✔	✔	✔	✔	✔
Visual analogue scale for dyspepsia	✔								✔							✔
Nepean Dyspepsia Index – Korean		✔							✔							✔
Functional Dyspepsia-Related Quality of Life		✔							✔							✔
EuroQol-5 Dimensions		✔							✔							✔
Functional Dyspepsia-related costs		✔							✔							✔
Beck’s Depression Inventory		✔							✔							✔
The State-Trait Anxiety Inventory		✔							✔							✔
Blood collection for ghrelin analysis		✔							✔							
Adverse events investigation^‡^		✔	✔	✔	✔	✔	✔	✔	✔	✔	✔	✔	✔	✔	✔	✔
Concomitant therapy evaluation		✔	✔	✔	✔	✔	✔	✔	✔	✔	✔	✔	✔	✔	✔	✔

± 1 day interval was allowed for each visit.

*8-week indicates 4 weeks after the 4-week acupuncture treatment.

^†^Adequate relief data will be collected by a telephone interview from 5 to 7 weeks for the acupuncture group.

^§^Adequate relief data will be collected by a telephone interview from 1 to 4 weeks for the waitlist group.

^‡^Adverse event data for the waitlist group will be collected from 5 to 8 weeks.

#### Primary outcome

The primary outcome is the proportion of responders (PR) [30], which is the proportion of subjects who answer “yes” to more than half of adequate relief questions in the treatment period. Participants are asked to answer the following question at each visit during the treatment period (twice weekly for 4 weeks), at each telephone visit during the waiting period, and at the follow-up visit: “After the last visit, have you had adequate relief of your stomach pain or discomfort?” Responders are defined as participants reporting adequate relief for at least 50% of the study period, i.e., responding “Yes” more than four times out of eight. The proportion of responders will be compared between the acupuncture group and the waitlist group.

#### Secondary outcomes

##### Nepean Dyspepsia Index – Korean version (NDI-K)

The Nepean Dyspepsia Index (NDI) [31,32] is a reliable and valid measure of QoL in FD. The NDI originally contains 42 items designed to measure symptoms and health-related QoL in dyspepsia. We will use the Korean version of NDI (NDI-K), as validated in 2004 by Lee et al. [33], consisting of two sections about symptom-based questions and QoL. In this trial, symptom-based questions such as period, severity, and degree of distress of 15 symptoms will be evaluated at baseline, 4 weeks, and 8 weeks.

##### EuroQol-5 Dimensions (EQ-5D)

EuroQol-5 Dimensions (EQ-5D) [34,35] is one of the standardized preference-based instruments as a measure of health-related QoL. This self-report questionnaire consists of an EQ-5D index and an EQ-5D VAS. To assess the index-based values (utilities), the three-level version of EQ-5D on five dimensions (mobility, self-care, usual activities, pain/discomfort, and anxiety/depression) will be used. EQ-5D VAS records an individual’s rating for his/her current health-related QoL state on a standard vertical 20 cm VAS (similar to a thermometer) where the endpoints are labelled as ‘best imaginable health state’ at the top and ‘worst imaginable health state’ at the bottom, respectively. A validated Korean version of EQ-5D will be administered in our study [36]. This measurement will be carried out at baseline and after 4 and 8 weeks.

##### Costs

FD-related costs will be assessed using medical records and a pre-defined questionnaire to collect direct medical cost including clinic or hospital care and prescription or over-the-counter drugs, and direct non-medical costs including formal/informal care and transportation costs. Time lost for doctor visits and absence from paid work due to FD will be taken from patients’ self-reported questionnaires. Direct medical costs used in this study, such as acupuncture costs, will reflect the real world situation in Korea from a societal perspective. Additional resource consumption data from patients will be measured at baseline and after 4 and 8 weeks.

##### Functional Dyspepsia-Related Quality of Life (FD-QoL) questionnaire

The FD-QoL questionnaire is composed of four categories of diet (five items), daily activity (four items), emotion (six items), and social functioning (six items). Higher total sum scores of FD-QoL indicate worse QoL. The questionnaire to be used in this study was validated among Korean FD patients in the previous study [37] and the measurement will be done at baseline, 4 weeks, and 8 weeks.

##### Beck’s Depression Inventory (BDI)

The Beck’s Depression Inventory (BDI) [38] is one of the most widely used instruments for measuring the severity of depression. This is a self-report inventory and consists of 21 multiple-choice questions. The participants will complete the BDI at baseline, 4 weeks, and 8 weeks.

##### The State-Trait Anxiety Inventory (STAI)

The State-Trait Anxiety Inventory (STAI) [39] is a psychological inventory to evaluate the degree of anxiety based on a 4-point Likert scale and consists of 40 items on a self-report basis. The STAI measures two types of anxiety – state of anxiety (20 items; anxiety triggered by a specific event) and trait of anxiety (20 items; anxiety derived from personal characteristic). Higher scores are positively correlated with higher levels of anxiety. This measurement will be completed at baseline, 4 weeks, and 8 weeks.

##### Ghrelin measurement (total ghrelin and deacylated ghrelin)

Ghrelin is mostly secreted by endocrine cells in the oxyntic mucosa of the stomach [40,41] and is present in the peripheral circulation as two subtypes: acylated and unacylated [42]. Its physiologic functions include regulating food intake [43,44] and secretion of gastric acid [45], accelerating gastric emptying [46], and stimulating gastric motility [47,48]. In our study, approximately 6 mL of whole blood, 3 mL each for total and unacylated ghrelin, will be collected on an empty stomach at baseline and at 4 weeks. The total and unacylated ghrelin assay will be performed using the commercially available radioimmunoassay and enzyme-linked immunosorbent assay kits.

### Sample size calculation

Sample size was determined based on our clinical experience and previous reports where the waitlist group achieved approximately 28% of the PR in patients with irritable bowel syndrome [49]; in our study, we expected that 60% of the participants on the acupuncture arm would achieve adequate relief for over a half of the study period while up to 25% of those on the waitlist arm would achieve such an outcome. Generally, a level of significance of α?=?0.05 and a power of 1–β?=?0.80 were used. The sample size in this trial was estimated according to the following formula where (p_t_?+?p_c_)/2:

$$\begin{array}{l} n=\;{\left(Z_{a/2}\sqrt{2\bar{p}\left(1-\bar{p}\right)}+\;\mathrm{Z\beta}\sqrt{p_{t}\left(1-p_{t}\right)+p_{c}\left(1-p_{c}\right)}\right)}^{2}\\ \;/{\left(p_{t}-\;p_{c}\right)}^{2} \end{array}$$

Assuming p_t_?=?0.6 (p_t_: the effect of the acupuncture group) and p_c_?=?0.25 (p_c_: the effect of the waitlist group), a sample size of *n*?=?30.1 is calculated to achieve 5% of significance level and 80% power. Considering an assumed dropout rate of 20%, a total of 76 participants will be needed with 1:1 allocation to each group (38 participants per group). First, both centers will respectively recruit 30 patients each. After that, the remaining 16 patients will be recruited and randomized competitively to include 76 patients in total.

### Statistical analysis

A statistical analysis will be performed by an independent statistician who is blinded to group allocation using SPSS 21.0 (IBM SPSS Statistics, New York, USA). Our independent statistician will have an access to the final dataset which will be kept locked so that investigators will have limited access. All data will be presented as means and standard deviations or number (%), and all analyses will be based on an intention-to-treat principle. For intention-to-treat analysis, the last observation carried forward rule will be applied. The statistical significance level will be set at 0.05 (two-sided), with the 95% confidence intervals.

#### Description of baseline characteristics and homogeneity of the two groups

For the description of baseline characteristics, the mean with standard deviation or range with minimum and maximum for continuous data and frequency with percentage for dichotomous data will be reported. For the homogeneity test of the baseline characteristics between the two groups, a two-sample *t*-test for continuous data and a *χ*^2^ test for dichotomous data will be performed. If there are baseline characteristics showing statistical significance between groups, analysis of covariance (ANCOVA) or logistic regression will be used for analysis and adjustment of baseline characteristics.

#### Primary outcome

The primary outcome, PR at 4 weeks, will be compared between groups using the *χ*^2^ test or logistic regression.

#### Secondary outcomes

Repeated measure two-factor analysis will be used to analyze the difference and mean change among baseline, 4 and 8 weeks, difference and mean change between groups, and interaction between groups and observed time. For dichotomous outcomes, the *χ*^2^ test or logistic regression will be used. For the comparison of adverse events (AEs) between groups, the *χ*^2^ test or Fisher’s exact test will be performed.

#### Data management

To promote data quality, the data will be collected by well-trained assessor and the double entry of the data will be implemented by clinical research coordinators.

### Safety

We will perform the following tests on all participants at screening: white blood cell count, hemoglobin, hematocrit, platelet, aspartate aminotransferase/alanine aminotransferase, gamma-glutamyl transpeptidase, blood urea nitrogen, creatinine, and erythrocyte sedimentation rate. These tests will help us to exclude participants who have serious diseases and abnormal liver, heart, kidney, or other organ function.

Participants will be asked about AEs at each clinic visit and during the 4-week telephone interviews. AEs will be defined as any unexpected and unfavorable signs, symptoms, or diseases temporally associated with acupuncture treatment. If any AEs occur, the appropriate treatment will be provided to the participant immediately. During the entire study, AEs will be recorded in the dedicated document including its severity and causality which will then be reviewed by the independent monitor.

Any serious AEs that are life-threatening or result in hospitalization, death, or significant or persistent disability will be promptly reported to the clinical research associate within 24 h from the time of recognition.

### Quality control

As we do not expect serious safety concerns, outstanding benefit, or futility in this study, no official Data Monitoring Committee will be established in each hospital. Instead, to retain the accuracy and quality of the clinical trial, the clinical research associate will conduct regular monitoring by checking investigator study files, informed consent forms, case report forms, compliance with treatments, serious AEs, and data records.

### Ethical approval and registration

This trial will be carried out according to the standards of the International Committee on Harmonization on Good Clinical Practice and the revised version of the Declaration of Helsinki. Institutional review boards at Kyung Hee University Korean Medicine Hospital and Kyung Hee University Hospital at Gangdong, have approved an initial version of this protocol (KOMCIRB 2013–05 for Kyung Hee University Korean Medicine Hospital and KHNMC-OH-IRB 2013–006 for the Kyung Hee University Hospital at Gangdong) on 28 June and 29 July 2013, respectively. This trial is registered in the ClinicalTrials.gov (NCT01921504). In addition, all investigators were trained to maintain the personal information of study participants and signed a pledge to protect the confidentiality of participants.

## Discussion

To our knowledge, acupuncture treatment for FD has not been intensively investigated. This article describes the protocol of a randomized trial with a waitlist control group to evaluate the effectiveness of usual acupuncture practice for FD patients in Korea. We expect that the results will be able to answer whether acupuncture treatments help adequate symptom improvement and quality of life, and whether ghrelin secretion is associated with acupuncture treatment, compared with the no acupuncture group.

A placebo control group is essential to determine the efficacy of a new treatment. Given that there exist some published sham-controlled trials of acupuncture for FD [4,19], some may argue that a sham acupuncture control should be adopted to investigate specific effect of acupuncture. However, it is yet to be clearly understood what specific components of acupuncture treatment work in the perceived therapeutic effect of acupuncture [50]. Sham controls used in the previous studies included superficial needling at non-acupuncture points with [4] or without electrical stimulation [19]. In our view, none of them are inert, and we could not find any rationale for a better choice of sham control for our study. Non-pharmacological intervention studies pose particular challenges to identify inactive control groups that generate expectancy comparable to the study intervention [20]. Thus, participants in the non-pharmacological intervention studies are often randomly allocated to waitlist control condition as a comparison to the treatment modality of interest. In this context, we decided to conduct a waitlist controlled trial as a starting point. From this trial, we can investigate how acupuncture might work against a group without acupuncture treatment. As natural fluctuations of symptoms in the disease process and high placebo response are unique challenges in FD trials, we can also obtain valuable information for further studies on how acupuncture treatment will affect symptoms of FD during the treatment and over a month of follow-up against a waitlist control. Additionally, a minimum treatment duration of 4 weeks that reflects the symptom periodicity is recommended for a treatment trial for functional gastrointestinal disorders [20]. Therefore, this will be the first Korean randomized controlled trial testing the adequate acupuncture intervention in terms of duration and reflecting everyday practice.

One of the crucial limitations of this study is that the doctors and participants cannot be blinded to the group allocation. Blinding refers to keeping key persons, such as participants, healthcare providers, outcome assessors, and data analysts, unaware of the treatment administered or of the true hypothesis of the trial [51,52]. Although all outcome measures will be administered and collected by the blinded nurse to minimize the risk of detection bias, and a statistician blind to group assignment will perform analysis of the data, the problem of participant unblinding still remains. We will carefully discuss residual sources of bias and their potential impact on clinical outcomes when we analyze the study findings and prepare for publication.

Some may argue that selection of ghrelin as a target mechanism may provide limited evidence. We understand that a variety of factors have been suggested to be associated with pathophysiology of FD [24-26]. Among many, ghrelin has been shown to play an important role in gastric motility, food intake, and potential gastroprotection against acute gastric mucosal injury [25,26]. However, to our best knowledge, there is no report on how ghrelin is related to acupuncture treatment for FD. Changes in ghrelin level after acupuncture, therefore, may not completely explain how acupuncture might work for symptom improvement in patients with FD. Nevertheless, ghrelin can be one of the many possible factors underlying the mechanism of acupuncture for FD and this study will help add evidence on this.

## Trial status

This study is currently recruiting participants. No interim analyses are planned and the primary results will be published by 2015.

## Abbreviations

AEs: Adverse events; AMSRC: Acupuncture and Meridian Science Research Center; BDI: Beck’s depression inventory; CV: Conception Vessel; EQ-5D: European quality of life-5 dimension; EX-HN: Extra acupoint of Head and Neck; FD: Functional dyspepsia; FD-QoL: Functional dyspepsia-related quality of life; GB: Gallbladder; LI: Large Intestine; NDI-K: Nepean dyspepsia index – Korean version; PC: Pericardium; PR: Proportion of responders; QoL: Quality of life; SI: Small Intestine; ST: Stomach; STAI: State-trait anxiety inventory; STRICTA: STandards for Reporting Interventions in Clinical Trials of Acupuncture; VAS: Visual analogue scale.

## Competing interests

The authors declare that they have no competing interests.

## Authors’ contributions

GH: Conception and design, data collection and analysis, manuscript writing, critical revision, and final approval of the manuscript. SJK: Conception and design, data collection and analysis, critical revision and final approval of the manuscript. JWP: Conception and design, data collection and analysis, critical revision, and final approval of the manuscript. JK: Data collection and analysis, critical revision and final approval of the manuscript. IY: Statistical design, data analysis and interpretation, critical revision and final approval of the manuscript. HJL: Conception and design, financial support, critical revision and final approval of manuscript. SYK: Design, data analysis and interpretation, critical revision and final approval of the manuscript. HSL: Conception and design, data analysis and interpretation, manuscript writing, critical revision, financial support, and final approval of the manuscript. All authors read and approved the final manuscript before submission.
